# A case of lip edema caused by the accidental ingestion of a bar of soap

**DOI:** 10.1002/ccr3.4484

**Published:** 2021-07-21

**Authors:** Tatsuya Fujihara, Ryosuke Ishida, Yuji Yamamori

**Affiliations:** ^1^ Division of Emergency and Critical Care Department Shimane Prefectural Central Hospital Izumo Japan

**Keywords:** accidental ingestion, lip edema, soap

## Abstract

The most common symptom following the accidental ingestion of a soap is lip edema. Although most cases are asymptomatic or exhibit mild symptoms, in some cases, aspiration pneumonia, oropharyngeal edema, and bronchial obstruction may be fatal.

## Graphical abstract

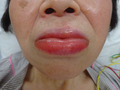

## 1 CASE

**FIGURE 1:**
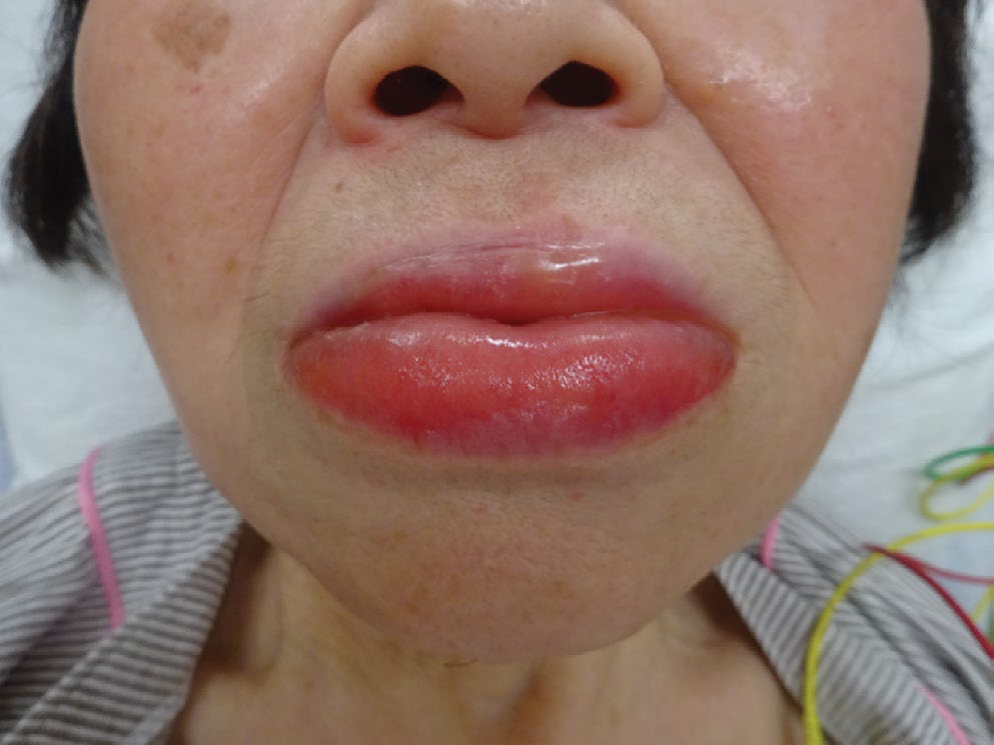
Lip edema on admission

An 83‐year‐old woman with Alzheimer's disease presented with lip edema and dyspnea 1 h after accidentally ingesting a bar of soap. Although she complained of slight dyspnea, her respiratory rate was 24 breaths/min and O_2_ saturation was 96% in room air. Physical examination revealed lip edema (Figure 1), but no stridor or obvious abnormalities in breath sounds. The patient was admitted for observation of her airway, respiratory, and digestive symptoms after administration of antihistamines but was discharged 12 h after the mishap, with no symptoms.

## 2 QUESTION

What is the most common symptom following the accidental ingestion of a bar of soap?

Most body soaps have an alkaline pH (9–12), which damages tissues by saponifying the fats.^1^, ^2^ A study by De Pralormo et al. revealed that lip edema is the most common symptom (55% of symptomatic patients), and dementia is the most common cause of accidental ingestion (39.8% of occurrences). In most cases, the symptoms of accidental ingestion of soap are mild. If accidental ingestion of a bar of soap occurs, patients' airway and respiratory conditions should be carefully monitored, as the soap may cause aspiration pneumonia, oropharyngeal edema, vomiting, or bronchial obstruction and may even be fatal, as reported previously.^1^

## DATA AVAILABILITY STATEMENT

Data available on request from the authors.

## CONFLICT OF INTEREST

Not declared.

## AUTHOR CONTRIBUTIONS

TF contributed to the clinical management of the patients, wrote the first draft, and managed all the submission processes. RI contributed to the clinical management of the patient and revised the manuscript. YY organized the manuscript.

## INFORMED CONSENT

Informed consent has been obtained for the publication of this clinical image.
